# Workforce interventions to improve access to emergency contraception pills: a systematic review of current evidence in low- and middle-income countries and recommendations for improving performance

**DOI:** 10.1186/s12913-015-0815-2

**Published:** 2015-04-26

**Authors:** Angela Dawson, Nguyen-Toan Tran, Elizabeth Westley, Viviana Mangiaterra, Mario Festin

**Affiliations:** 1 World Health Organization Collaborating Centre for Nursing, Midwifery and Health Development, Faculty of Health, University of Technology, Sydney (UTS), Jones Street, Sydney, NSW Australia; 2 School of Public Health and Community Medicine, University of New South Wales, High St, Sydney, 2052 Australia; 3 International Consortium for Emergency Contraception, 45 Broadway, New York, USA; 4 RMNCH and HSS Technical Advice & Partnerships Department The Global Fund to Fight AIDS, Tuberculosis and Malaria, Chemin de Blandonnet 8, 1214 Vernier, Geneva Switzerland; 5 Department of Reproductive Health and Research, World Health Organization, Avenue Appia, Geneva, Switzerland

**Keywords:** Emergency contraception pills, Workforce, Low and lower-middle income countries, Access to contraception

## Abstract

**Background:**

Emergency contraceptive pills (ECP) are one of the 13 essential commodities addressed by the UN Commission on Life-Saving Commodities for Women and Children. Although ECP have been available for 20 years, a number of barriers still limit women’s access ECP in low and middle-income countries (LMIC). The workforce who prescribe or dispense ECP are diverse reflecting the varied contexts where ECP are available across the health, commercial and justice sectors and in the community. No reviews currently exist that examine the roles and experiences of the workforce that provide ECP in LMIC.

**Method:**

We present a narrative synthesis of research to: identify provider factors that facilitate and constraint access to ECP; assess the effectiveness of associated interventions and; explore associated health system issues in LMIC. A search of bibliographic databases, meta-indexes and websites was undertaken to retrieve peer reviewed and grey literature. Literature was screened and identified documents examined to appraise quality.

**Results:**

Thirty-seven documents were included in the review. Studies focused on formal health workers revealing knowledge gaps concerning the role of private sector and non-health providers who increasingly provide ECP. Data from the findings section in the documents were coded under 4 themes: provider knowledge; provider attitudes and beliefs; provider practice and provider training. The analysis revealed provider knowledge gaps, less than favourable attitudes and practice issues. The findings provide limited insight into products prescribed and/or dispensed, the frequency of provision, and information and advice offered to consumers. Pre and in-service training needs were noted.

**Conclusion:**

As the provision of ECPs shifts from the clinic-based health sector to increasing provision by the private sector, the limited understanding of provider performance and the practice gaps revealed in this review highlight the need to further examine provider performance to inform the development of appropriate workforce interventions. A standardized approach to assessing performance using agreed outcomes measures may serve to ensure a systematic way forward that is inclusive of the diverse workforce that deliver ECP. Recommendations are outlined to enhance the performance of providers to improve access to ECP. A framework is offered to help guide this process with indicators.

## Background

Universal access to sexual and reproductive health and rights is a necessary part of healthy societies. Globally, 222 million women who want to prevent pregnancy are not accessing effective, modern methods of contraception. As a result, each year there are approximately 86 million unplanned pregnancies, 33 million unplanned births [1] and 20 million unsafe abortions [2]. Adolescent women aged 15–19 years give birth to 15 million babies each year with over 90% in low and middle-income countries (LMIC) [3]. Complications from pregnancy and birth are among the leading causes of death for young women [4] and have been linked to poor access to health services, information and care [5]. A competent, motivated and well-managed workforce, as part of a robust health system, is essential to the delivery of evidence-based packages of care for women to improve reproductive and maternal health outcomes [6,7].

Emergency contraceptive pills (ECP) provide women with a safe and fairly effective opportunity to prevent pregnancy after unprotected intercourse. ECP is one of the 13 essential commodities that are addressed by the UN Commission on Life-Saving Commodities for Women and Children (UNCoLSC) [8]. Despite being available for nearly 20 years, a number of barriers still limit women’s access to ECP [9]. The workforce who prescribes or dispense ECP are diverse and reflect the varied contexts where ECP is available across the health, commercial and justice sectors, as well as in the community. In both the public and private health sector at the primary, secondary and tertiary levels, doctors, pharmacists, nurses, midwives and paramedical staff such as nursing assistants can provide access to ECP. At the community level, formal and lay health workers distribute contraceptives. In Bangladesh for example, family welfare assistants supply contraceptives during home visits and volunteer female community health workers (CHW), who reside in rural villages and urban neighbourhoods distribute condoms and contraceptive pills to women [10]. As part of a global trend to move ECP from a prescription to an over the counter (OTC) product, the commercial private sector is playing an increasingly important role. ECP are widely available in pharmacies [11] and sold by pharmacists and retail staff also known as drug vendors or patent medicine vendors [12]. Other providers in the commercial sector include drug sellers such as patent medicine vendors or retailers in Nigeria who have no formal pharmacy training and sell pharmaceutical products for profit. These providers are usually the primary source of drugs, particularly for the poor [12]. Other providers have been trained to distribute ECP including the police in situations where women have been sexually assaulted [13].

Insight into the availability of these providers, their practice and the challenges they face may offer an understanding of the factors that influence access to and demand for ECP. However the diversity of these providers does not lend itself to current measures of access to workforce and therefore contraceptive coverage because measures such as the Health Workers’ Reach Index (HWRI) or health worker density ratio focus on doctors, nurses, and midwives. These measures ignore the roles other providers play in providing access to ECP and the complexity of the situation can be illustrated by a capacity project study where African countries that have the same HWRI scores can have different contraceptive prevalence rates [14]. No systematic review currently exists that examines the roles and experiences of this diverse workforce that provide ECP in LMIC.

In order to provide evidence to contribute to strengthening ECP delivery channels and developing policy guidance and tools to expand access to ECP particularly among vulnerable groups, we undertook a narrative synthesis of current research focusing on providers of ECP. The aim of this review was to identify workforce opportunities and interventions in LMIC that can facilitate increased access to ECP. The results of this review and related reviews into consumer and service delivery experiences will provide the necessary evidence to assist the ECP Technical Reference Team to help carry forward UNCoLSC recommendations at the global and national levels [15].

## Methods

The bibliographic databases (CINAHL, MEDLINE, PubMed, SCOPUS, ProQuest Health & Medical Complete, Web of Science, African Journals On Line), meta-Indexes (Popline, Eldis knowledge services, Reproductive Health Library) and websites of relevant organizations (The Guttmacher Institute, The International Consortium on Emergency Contraception, Population Council) were searched. The mixed nature of the methodologies of studies identified meant that results could not be combined. As a result a narrative synthesis methodology [16] was applied to analyse research papers and reports whose quality has been appraised using acknowledged tools.

**Figure 1:**
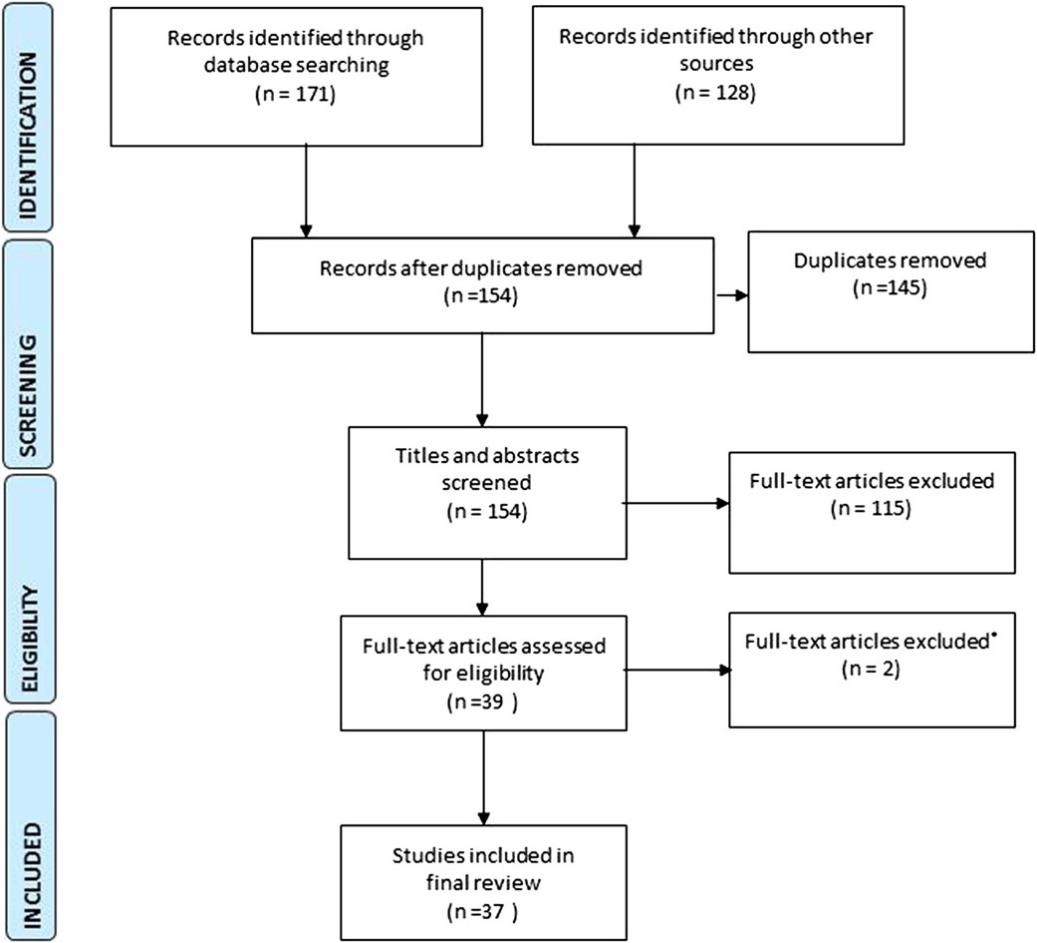
Preferred reporting diagram for systematic reviews and meta-analyses (PRISMA) showing selection of publications for review.

A Population, Interventions, Comparators, Outcomes, Study design (PICOS) question was formed to guide this review as per guidelines [17]. The review question was: What provider factors facilitate and constrain access to ECP in LMIC? Outcomes of interest include: provider knowledge; training outcomes, and practices. The review aimed to source descriptive as well as intervention studies that shed light on access to ECP. Observational studies, quasi experimental and non-experimental descriptive studies were considered suitable for inclusion and a systematic search of the primary research literature published from 2003 to 2013 in English and in LMIC was undertaken. Electronic databases and the internet were searched using the Medline Medical subject headings (MeSH) ‘Postcoital Contraception’ and’Health Services Accessibility’ and ‘Healthcare Delivery’ and ‘Contraceptive Distribution’ and ‘Health Manpower’ or ‘Health Personnel’ and ‘Developing Countries’ and supplemented by the terms key words ‘emergency contraception’, or ‘emergency contraceptive pills’ and ‘provider’ or ‘workforce’ or ‘nurse’ or ‘doctor’ or pharmacist’ using the MeSH terms, abstract and keyword options. For example, a search of ProQuest Health & Medical Complete using the terms ft(emergency contraception) AND ft((provider OR nurse)) AND ft((doctor OR pharmacist)) AND mesh (Postcoital Contraception) peer reviewed documents in English 2003–2013 returned 47 documents. The Preferred Reporting Items for Systematic Reviews and Meta-Analyses (PRISMA) guidelines were used to report the review process [18] (see Figure 1). Duplicate records were removed and the remaining records then screened using the PICOS question. Papers without data, those older than 10 years or whose focus was outside of the aim were removed. In the example of the ProQuest Health & Medical Complete database search mentioned above two of the 47 papers were included in the review as their focus was on providers in LMIC and within the date limitations. A similar process was undertaken with the other searches and relevant papers compiled in an Endnote bibliographic database.

### Appraisal

Thirty-nine papers were identified as relevant. These papers were appraised by all authors for their quality using the Critical Appraisal Skills Programme (CASP) tool for qualitative research [19] and Pluye et al.’s [20] scoring system was used to assess the non-experimental studies and mixed methods. Research reports were classified as grey literature and were appraised using a checklist developed for this purpose [21]. Two papers were excluded due to the lack of data on ECP [22,23]. Ethical approval was not required for this study as all data is in the public domain.

### Data extraction and synthesis

A narrative synthesis approach was undertaken according to Popay [16] allowing qualitative and quantitative data to be examined. The results sections of each of the 37 papers were analysed to identify evidence where providers had helped to increase to ECP in LMIC. A thematic analysis was conducted by the first author using tables and discussed with other authors in order to reach consensus. The relationships within and between studies were explored and coded using QSR NVivo 10 software. The analysis was guided by the World Health Organization (WHO) Health System [24] Building Blocks, a framework that describes the six components of health systems, namely service delivery, health workforce, health information, medical technologies, health financing, and leadership and governance. Concept maps were used to plot patterns and relationships across the themes and subthemes and robustness assessed through critical reflection and discussion among the authors.

## Results

Of the 37 papers that were included in review providers included groups of clinically-based health professionals, such as doctors or nurses in hospitals, and health professionals and service staff in retail contexts such as pharmacists and sales people or drug vendors in private pharmacies or shops (see Table 1 for an outline of all provider types in the studies included in this review). Nine studies focused on those working in retail settings, i.e. pharmacist and/or drug vendors, 26 focused on clinic or hospital-based personnel (doctors, nurses) and only two included police.

**Table 1:** **Provider types in the papers included in the review**

Reference	Provider type						
	Pharmacist	Drug vendor	Dr	Nurse	Paramedic	CHW	Police
(Abdulghani, Karim & Irfan 2009)			✓				
(Ahonsi et al. 2012)	✓	✓	✓	✓			
(Balakumar 2013)	✓						
(Bibi et al. 2013)			✓				
(Byamugisha et al. 2007)			✓	✓	✓		
(Chowdhury 2013)			✓				
(Creanga et al. 2011)			✓	✓	✓		
(Dawit, Van der Merwe & Smith 2010)	✓	✓					
(Ebuehi, Ebuehi & Inem 2006)	✓		✓	✓		✓	
(Ehrle & Sarker 2011)	✓	✓					
(Fayemi et al. 2010)		✓					
(Gawade et al. 2009)	✓				✓		
(Geidam, Kullima & Sadiq 2010)	✓		✓	✓	✓		
(Ibrahim, Ahmed & Shaaban 2013)			✓				
(Judge, Peterman & Keesbury 2011)			✓	✓	✓		
(Kassa, Hiwot et al. 2009)			✓				✓
(Kassaye & Dwizedi 2009)			✓				
(Kestler & Ramirez 2004)			✓				✓
(Khan et al. 2012)			✓	✓	✓		
(Kishore, Misro & Nandan 2010)	✓		✓	✓	✓		
(Lemma 2009)	✓						
(Liambila, Obare & Keesbury 2010)	✓						
(Mané et al. 2012)			✓	✓	✓		
(Mayhew, Osei & Bajos 2013)			✓	✓	✓		
(Mir & Malik 2010)						✓	
(Mishra & Saxena 2013)							
(Mondal et al. 2006)			✓				
(Obare, Keesbury & Liambila 2009)					✓		
(Okonofua et al. 2009)			✓				
(Onwuhafua et al. 2005)					✓		
(Oriji & Omietimi 2011)			✓				
(Shaki 2013)			✓	✓	✓		
(Syahlul & Amir 2005)			✓				
(Sychareun et al. 2010)			✓	✓			
(Thapa 2013)				✓			
(Tripathi, Rathore & Sachdeva 2003)			✓	✓	✓		
(Worku & Teklu 2011)							

Geographically, the papers were concentrated in one region: twenty one of the papers were based on studies carried out in Africa (including seven from Nigeria and five from Ethiopia). Fourteen studies were carried out in Asia, with eight of these conducted in India. Two studies in the review were undertaken in Central America.

Descriptive survey designs underpinned the majority of the studies (n = 30) (See Table 2 for a summary of all papers in the review). Data from the findings section in the documents were coded under four themes: provider knowledge; provider attitudes and beliefs; provider practice and provider training.

**Table 2:** **37 documents grouped under ECP providers**

Reference	Context	Method	Sample/ participants	Aim	Findings
(Abdulghani, Karim & Irfan 2009)	Hospital Karachi, Pakistan.	Descriptive quantitative survey	45 faculty physicians (33%), residents (27%), medical officers (40%), 36% male and 64% female physicians; of them, the majority (64%) were married	To assess the knowledge of family medicine providers and their attitudes towards ECP	71% familiar with ECP, 40% prescribed in last year. Barriers noted ‘it will promote promiscuity’ (31%), religious/ethical reasons (27%), liability (40%), teratogenicity (44%), and inexperience (40%). 38% incorrectly chose menstrual irregularity as the most common side-effect, 33% answered that EC was not an abortifacient while 42% were unsure.
(Ahonsi et al. 2012)	Nigeria: Kaduna and Abuja.	Descriptive quantitative survey and qualitative interviews	407 providers: pharmacists (36%), followed by PMVs (30%), hospital and clinic staff private (22%), public sector (12%).	To document EC-related knowledge, attitudes, and practices among EC providers and opinion leaders	Providers: knowledge gaps, number of factors facilitated positive KAP.
			13 stakeholders: government, professional associations, pharmaceutical companies religious leaders and NGOs		Public sector stakeholders less conversant with ECP than NGOs, Govt must take greater role in policy, procurement, management and monitoring
(Balakumar 2013)	Nepal: Kathmandu, Lalitpur, and Bhaktapur	Descriptive qualitative interviews content analysis	22 pharmacists	To examine pharmacists’ levels of knowledge surrounding EC and MA and their attitudes towards the use of these methods by adolescents	Generally supportive, more rural pharmacist more conservative attitudes
(Bibi et al. 2013)	India Hyderabad	Descriptive quantitative survey	270 general practitioners	To examine impact of physician ECP training	31.1% attended 5 days training course on FP in the past, 69.9% did not have any training. Source of training: government institutes 17%, NGO 14.1%. Significant positive difference was noted on EC knowledge, attitude and use in group who attended family planning training
(Byamugisha et al. 2007)	Uganda: Kampala	Descriptive quantitative survey	247 providers Midwives/nurses 74.9%, Clinical officer 6.5%, Doctor (medical officer) 10.9%, Gynaecologist 4.5%, Other.2%	To assess provider EC knowledge, attitudes and prescribing pattern	80% had knowledge of ECP, 25% was not sure about the time limit within which EC is effective, and 50% of the participants had obtained information from a physician (26.4%) or from a training school (24%). The Yuzpe regimen most commonly mentioned and prescribed method of EC. The HCWs attitudes to EC were generally positive, and the community should be sensitised about EC, significant difference between having had a family planning educational update or not in the last year and knowledge of EC (p = 0.005)
(Chowdhury 2013)	Kerala, India	Descriptive cross sectional survey	106 randomly selected Obstetrics and Gynaecology professionals	To develop and validate an Scale for measuring attitudes towards providing of MTP/ECP services and examine the influence of socio-cultural identities on these attitudes	Up to 60% of the OGPs had negative attitudes towards MTP/ECP provision and this is reflected in their practice that makes MTP/ECP services less accessible for women in many institutions. Personal beliefs and sector of worksite shape the stated attitudes of providers.
(Creanga et al. 2011)	Ghana: Kumasi	Descriptive quantitative survey	600 providers	To assess the theoretical and practical knowledge about EC among (FP providers	FP providers gave 4.1 correct answers to the 11 questions assessing theoretical knowledge and 5.6 correct answers to the 8 questions assessing their practical ability to provide EC. The health sector in which FP providers worked, their education and having received EC-specific training, the number of services offered, and the number of women seen during a week were all significant correlates of both theoretical and practical knowledge about EC. The 2 knowledge domains were significantly and positively associated.
			75% providers worked in pharmacies and 83.7% in the private sector		
(Dawit, Van der Merwe & Smith 2010)	Ethiopia: Addis Ababa	Descriptive quantitative survey	40 service providers	To determine pharmacists’ and drug vendors’ levels of knowledge, and attitudes towards and practices of ECP	Lack of knowledge about side-effects, contra-indications, and types of ECs. Most portrayed subjective attitudes towards easy EC access, especially for adolescent girls, since they believed that it would encourage promiscuity and unprotected sexual intercourse.
			Pharmacists’ and drug vendors’		
(Ebuehi, Ebuehi & Inem 2006)	Nigeria: Lagos State	Descriptive quantitative survey	256 health care providers	To assess provider knowledge and practice	9/10 providers had heard of emergency contraception, 50% knew the correct time frame for effective use 75% knew that the pills prevent pregnancy; >30% believed that they act as an abortifacient. <30% who had heard of the pills knew they are legal in Nigeria. Of those who had heard about emergency contraception, 58% provided clients with emergency contraceptive pills, 10% of these providers could correctly identify the drug, dose and timing of the first pill in the regimen. < than 1/10 who knew of emergency contraception said they always provided information to clients, whereas ¼ said they never did so
			Physician 45.3%,Nurse 27.0%, Pharmacist 18.0%, Community health worker 9.8%		
(Ehrle & Sarker 2011)	Nicaragua: Managua,	Descriptive quantitative survey	93 pharmacy employees	To access pharmacy personnel’s knowledge and attitudes	100% knew about emergency contraceptive pills and reported experience selling them. 92% sold them at least once a week without a prescription (97%). 45% knew that the pills can be taken up to three days afterward; none knew that the pills are effective up to five days afterward. 39% thought the pills can induce abortion, and overestimated contraindications and side effects. 75% believed the availability of emergency contraceptive pills discourages use of ongoing methods (encourages sexual risk-taking (82%) and increases transmission of HIV and other STIs (76%), 68% thought emergency contraceptive pills are necessary to reduce unwanted and unplanned pregnancy; 65% were willing to provide them to all women in need, 13% would provide them to minors.
(Fayemi et al. 2010)	Nigeria: Oyo and Ogun States	Descriptive quantitative survey	97 Patent Medicine Vendor (PMV), (60.8 per cent female	To assess knowledge, dispensing practices of PMVs, and referral for ECP	27.8% of respondents were not aware of ECP, and only half knew that ECP could prevent pregnancy, 40% had ever dispensed ECP. Reasons proffered by those who do not dispense ECP included barriers from the State Ministry of Health, police, other regulatory agencies, and religious beliefs. Only 50.5 per cent have referral arrangements for clients
(Gawade et al. 2009)	India: Mahaeshtra	Descriptive quantitative pre and post survey	102 auxiliary nurse midwives, lady health visitors, health assistants, multipurpose workers, pharmacists	To assess training outcomes and feasibility of paramedical to dispense ECP	Knowledge and attitudes significantly improved as a result of the training programme.
(Geidam, Kullima & Sadiq 2010)	Nigeria: Borno State	Descriptive quantitative survey	physicians, pharmacist and nurse/midwives	To determine the knowledge, attitudes and provision of emergency contraception	80.6% awareness, lower among nurse/midwives (69.8%), 49.5% of the respondent aware of Oestrogen as a method and 5.1% knew that Danazol can be used for emergency contraception, 10.2% were not sure of the correct timing of EC, 36.2% of the respondents had ever provided EC before. Physicians were more likely to approve the use while pharmacists were more likely to have provided EC before.
(Ibrahim, Ahmed & Shaaban 2013)	Egypt: Ismailia,	Descriptive quantitative survey	270 health care providers (obstetrics and gynaecology specialists and general practitioners or family physicians)	To explore the knowledge, attitude and practice of health care providers	Knowledge of specialists was significantly higher than general practitioners/family physicians regarding the three most commonly used methods of EC, viz; combined oral contraceptive (Yuzpe) method, progesterone only pills (plan B) method and IUCD. Only 39.5% of specialists and 24.0% of GPs/family physicians had good knowledge of EC (p = 0.01). 45.7% of specialists and 42.6% of GPs/family physicians had favourable attitude toward EC with no significant difference. 39.5% of specialists and 26.6% of GPs/family physicians reported ever prescribing EC. Yuzpe method was the most commonly prescribed method by specialists (31.5%) and GPs/family physicians (27.0%) with no significant difference. Knowledge and favourable attitude were significantly associated in both groups. Age and years of experience significantly affected the three outcome measures
(Judge, Peterman & Keesbury 2011)	Kenya: Nairobi, Coast, Rift Valley, Nyanza and Western. provinces and Ethiopia: Addis Ababa, Amhara, Oromiya, Tigray Southern Nations, Nationalities and People’s Region	Descriptive quantitative survey bivariate analyses and multivariate logistical regression models	524 health care providers in 199 government health care facilities in Kenya	To gain and understanding of the impact of provider biases and gaps in provider knowledge on EC counselling	54% and 31% of Kenyan and Ethiopian providers, respectively, display strong EC counselling behaviour, while 61% and 55%, respectively, and report having ever provided EC. Bivariate and multivariate results show that, in Kenya, increased EC counselling and provision behaviours are associated with higher levels of provider knowledge.
			121 health care providers in Ethiopia nurses (73%),		
			followed by midwives (14%); 3 doctors		
(Kassa, Hiwot et al. 2009)	Ethiopia Addis Ababa, hospitals	Mixed methods- Descriptive Cross sectional survey of all health facilities in Addis Ababa to assess EC provision after sexual assault. In-depth interview were conducted with key informants at police stations.	576 health facilities in Addis Ababa	To examine the potential barriers to accessing ECP among sexual assault survivors	Five public hospitals and one model clinic give 1.04% of all facilities treatment to victims of sexual assault and provide ECP. No private hospital provides treatment. Low police knowledge of ECP and referral usually to model clinic. Lengthy processing times and cost to women make court action difficult
			4 police stations		
(Kassaye & Dwizedi 2009)	Ethiopia: Addis Ababa	Descriptive quantitative survey	445 physicians at government hospitals	To assess attitudes of physicians towards routine counselling and advance prescription	55.3% received a very good and good knowledge score. Physicians of ObGyn dept. were more knowledgeable than others (p < .0001.). Attitudes of physicians were favourable (64%) towards EC. Being a member of ObGyn dept. showed a very good knowledge score, and past counselling and prescribing EC had favourable attitudes. 72.4% have never prescribed EC. Physicians who ever counselled and prescribed EC before the survey were more likely to have a very good knowledge score (181 p < 0.0001), favourable attitudes (176 p < .001) and past prescribing (124 p < 0.15) than others. 83.6% agreed on the role of routine counselling and advance prescription supply of EC, in provision, promotion and information dissemination, and among them 68.8%, were willing to prescribe EC in the future. 90% had concerns like it might encourage repeated use. 84% mentioned mass media, printed materials, women organization and telephone including text messages and involvement of male partners as better options for EC advocacy, information dissemination, and provision.
(Kestler & Ramirez 2004)	Guatemala	Descriptive quantitative survey	120 people who completed ECP training physicians (20%), nurses (17%), public employees (police officers - 16%), and social workers (11%), other 37%	To examine the awareness of and access to emergency EC among the medical community	14% prior to the workshop had ever recommended EC. After the workshop, 62% reported having recommended EC. 14% reported having given an information talk, trained counsellors on EC, 8% gave talks or provided information in organizations different from their own, and only 4% replicated the workshop
(Khan et al. 2012)	India: Lucknow, Aligarh, Agra and Delhi in Uttar Pradesh	Descriptive quantitative survey and qualitative interviews	315 providers: Drs (83), pharmacists (199), others (33)	To document EC-related knowledge, attitudes, and practices among EC providers and opinion leaders	Despite providers saying safe and effective they were incorrectly informed about ECP’s mechanism of action. Pharmacists do not provide any information on ECP to customers, for fear of embarrassing customers due to lack of privacy in their shops, lack time for any real client counselling. Appropriate for married women and that a minimum age restriction of 18 to 20 should be instituted. Drs felt it should be prescription only. Opinion leaders supported access but had reservations about promoting or mainstreaming ECP
			19 Key stakeholders: MoH, professional associations, NGOs, donors		
(Kishore, Misro & Nandan 2010)	India: south district in Delhi,	Descriptive quantitative survey	428 providers 72 Lady Health Visitor/	To assess knowledge, attitude and dispensing practices of ECP	Medical officers were observed to be most knowledgeable about E-pills and the pharmacists were the least. The correct prescribed dose of E-pill was known only to 32% of the providers while 49% knew about its right time of intake. Misconceptions and apprehensions for promoting its use were very much prevalent even among medical officers as majority felt that open access to E-pills would increase promiscuity. The dispensing practice of providers was found positively (p < 0.05) correlated with their knowledge. Training resulted a significant (p < 0.05) improvement in knowledge, attitude and dispensing practice of the providers. Knowledge and training combined together contributed 35% to the dispensing practice (R^2^ = 0.35)
			Staff Nurse, 164 Auxiliary Nurse Midwife (ANMs) and 67 were Pharmacists		
(Lemma 2009)	Ethiopia: Addis Ababa	Descriptive quantitative survey	40 licensed pharmacists and drug vendors	To determine pharmacists and drug vendors' level of knowledge, attitude towards and practice on	Service providers were knowledgeable on the purpose and dosing schedule of EC, they lacked knowledge on side-effects, contra-indications, and types of ECs. Most respondents portrayed a subjective attitude towards easy EC access of especially adolescent girls, since they believed that it will encourage promiscuity and unprotected intercourse.
(Liambila, Obare & Keesbury 2010)	Kenya: Nairobi,	Experimental design baseline and endline assessments of EC provision through the use of mystery clients. Survey and qualitative interviews	9 intervention and control 8 pharmacies. Intervention pharmacies received weekly updates on EC, fliers with three key messages on EC, and information, education, and communication materials	To evaluate the provision of reproductive health information and services to users of ECs by private pharmacists	The differences between the control and intervention pharmacies with respect to the provision of additional information on EC and regular family planning services are in the expected direction but statistically insignificant. In contrast, the likelihood of providing information or referral for counselling or testing for sexually transmitted infections or HIV was lower in the intervention than in the control pharmacies but the difference was also not statistically significant.
(Mané et al. 2012)	Senegal: Dakar and Thiès urban areas	Descriptive quantitative survey and qualitative interviews	34 opinion leaders and 155 providers (doctors, midwives, nurses/ nurse assistants, pharmacists, and pharmacy counter staff)	To document EC-related knowledge, attitudes, and practices among EC providers and opinion leaders	Providers (38%) have not received any EC training. 41% of trained providers were comfortable with ECP sold without prescription, while the majority preferred tighter controls. preferred EC provision limited to conventional health facilities such as hospitals, health centres, private clinics, and pharmacies: They do not favour extending EC at community and school levels through other supply means; more than others, are at greater risk of acquiring STIs, have multiple sexual partners, and not use regular contraception; Over two-thirds of providers indicated providing instructions on ECP use. Few KOLs are informed about ECP’s mode of action; most are in favour of integrating EC in the national guidelines for FP service provision Legislation should be revised similarly to other contraceptive products: urged caution in expanding EC access to the community level.
(Mayhew, Osei & Bajos 2013)	Ghana and Burkina Faso	Qualitative interviews	31 Providers Nurse/midwife 18, Drs. 5, Pharmacists 4, Pharmacist assistant 4	To assess providers’ EC attitudes and delivery practices	Result on two dimensions reflecting providers’ “acceptance” and “provision” of EC. Provider attitudes broadly favoured EC, although most in Burkina Faso were cautious about providing it (fearing that regular use might displace condom use, thus increasing HIV risk), while in Ghana, many highlighted useful role of EC in reducing unwanted pregnancy. Overall, respondents wanted to limit distribution to health facilities and pharmacies and were reactive, rather than proactive, EC providers. Their attitude towards people seeking emergency contraception varied: those suffering contraceptive method failure or provider failure were seen as deserving, while those who came because they had used their contraceptive method incorrectly or not used one at all were regarded less favourably.
(Mir & Malik 2010)	Pakistan: Rawalpindi district	Descriptive quantitative survey	67 Lady Health Supervisor	To explore the ECP knowledge, attitudes and practices of the Lady Health Supervisor	Insufficient knowledge, high misinformation and strongly negative attitudes. > 50% did not know that ECP do not cause abortion. About 4/5 believed that emergency contraceptive pills will lead to ‘evil’ practices in society. > 4/5 recognized that the clients of National Program for Family Planning need ECP. The attitudes significantly associated with knowledge
(Mishra & Saxena 2013)	India: Delhi	Descriptive quantitative survey	60 pharmacies	To evaluate the knowledge and over-the-counter services provided by the pharmacists in	2 to 500 packs sold per month. 62% of the pharmacists claimed that majority of the clients repeated use during the same month, 18% of the clients were referred by doctors, and 82% directly approached the pharmacists. 1/3 clients were adolescents. 67% of the pharmacists had adequate knowledge about EC, 3.3% asked about the last menstrual period or the time elapsed since the last unprotected intercourse. No pharmacist asked if one or multiple unprotected acts of intercourse, if any regular contraceptive method was being used, or the reason for EC intake. 91.7% explained the dosage schedule to clients. 50% explained client may experience side effects. No pharmacists suggested STI screening, 35% counselled the clients regarding regular contraception.
(Mondal et al. 2006)	India Kolkata, RG Kar Medical College and in the district of 24 Parganas	Descriptive quantitative survey	140 healthcare providers and 480 beneficiaries	to evaluate the knowledge, attitude and practice	Providers and beneficiaries lacked knowledge of the concept of ECP. Paramedical providers were more likely to recommend ECP after unprotected sex and to prevent abortion than Gynaecologists and Obstetricians.
(Obare, Keesbury & Liambila 2009)	Kenya	Descriptive quantitative survey and qualitative interview	103 Mystery client visits to 20 randomly selected pharmacies with mystery clients acting out two scenarios: an experienced client, and a non-experienced client.	To assess pharmacists and others provider knowledge and practices regarding EC	Some providers insist on doctors’ prescriptions before they can dispense EC. There are variations among providers on the recommended dosage and possible side-effects of EC pills. MCs presenting as inexperienced clients were significantly more likely to be given additional information on EC than the experienced ones. There was no significant difference in the provision of additional reproductive health (RH) information/services by the scenario presented.
(Okonofua et al. 2009)	Nigeria from 11 States	Descriptive quantitative survey	174 private medical practitioners	To determine knowledge and practices of EC by private medical practitioners	79.9% of the respondents correctly described emergency contraceptive methods; only 23% reported that they had emergency contraceptives in their clinics, while only 13.8% of the practitioners used the correct brands and dosages of emergency contraceptives currently available in the country. Similarly, a large proportion of the practitioners did not know the exact timing of emergency contraceptives in relation to sexual intercourse, while only a few gave correct names and dosages of administration. The results show inadequate knowledge and poor use of emergency contraceptives by private medical practitioners in Nigeria
(Onwuhafua et al. 2005)	Nigeria: Kaduna State	Descriptive quantitative survey	232 community health extension workers	To determine knowledge, attitude and practice of family planning amongst community health extension workers	All could recall at least one modern method of family planning. The oral contraceptive pill (OCP) (85.8%), injectable contraceptives (85.3%), and the intra-uterine contraceptive device (IUCD) (56.0%), were most widely known about. Emergency contraception was not known about. A high percentage of female Chews have practised family planning: 115 (74.7%) have used at least one method and this is more among the married women. Methods ever used included injectable contraceptive (57.4%), OCP (47.0%), and IUCD (22.6%). Fifty percent of females were current users. Non-current users were likely to be between the ages of 25–29 years especially when not married, and 35–39 years when married. Reasons for the non-use of family planning by female Chews were side effects, not being married, not being sexually active and religious beliefs
(Oriji & Omietimi 2011)	Nigeria: Port Harcourt	Descriptive quantitative survey	100 medical doctors	To determine knowledge, attitude, and practice of emergency contraception among medical doctors	Knowledge about its use was poor. 98% of them were aware of emergency contraception, 58% could not identify correctly any type. Oral mifepristone (RU486) was the most recognized form of EC identified by 38% of the doctors. Rape would be the commonest indication for emergency contraception as reported by 76% of the doctors, ahead of missed pills by 36% and incestuous sexual intercourse by 46% of the doctors. Postinor (levonorgestrel) given within 72 hours and IUCD inserted within 5 days of intercourse were the commonest forms of EC administered by 26% each of the doctors interviewed. Conclusion: Although the awareness of EC is high among the doctors in Port Harcourt, the knowledge and use of EC is low.
(Shaki 2013)	Tanzania: Dar es Salaam	Descriptive quantitative survey with open questions thematically coded	268 health care providers and 300 medical students	To examine knowledge, attitude and practices of EC among health care providers and medical students	A lack of knowledge about EC was found as only 30.4% of the health care providers and 32.9% of the medical students were found to have adequate knowledge of EC. EC provision was reported by 31% of the providers and EC utilization was found to be 14.9% among medical students. Majority of health care providers (94.9%) and 90.7% of medical students had positive attitudes towards EC provision and utilization respectively. Awareness of emergency contraception among health care providers and medical students was found to be moderate (59% vs 53.7%). Despite this, adequate knowledge on emergency contraception on both groups was low (30.4% vs 32.9%). Provision of EC by the health care providers as well as utilization of EC among medical students was found to be low. The majority of the providers and students had positive attitudes towards EC practices i.e. provision among the health care providers as well as EC utilization among the medical students.
(Syahlul & Amir 2005)	Indonesia: Jakarta	Descriptive quantitative survey	205 general practitioners 142 obstetricians and gynaecologists in 36 Community Health Centres and 25 private clinics	To examine attitude of medical practitioners towards the availability of EC	Most participants were familiar with EC, only 22% received a very good knowledge score (4 or 5/5 answers correct), while 52% received a poor score (0-2/5 correct). Most participants did not support the OTC availability of EC (70%). Logistic regression identified that participants who prescribed EC had an Odds of 3.8 (95% CI 1.90, 7.73) of approving OTC EC, after adjustment for age and speciality
(Sychareun et al. 2010)	Laos	Qualitative methods in-depth interviews, content analysis	10 policy makers, 22 public providers, and 10 providers at private clinics	To examine policy maker knowledge and attitudes towards provision of ECP	Most policy makers and health providers knew about ECP and supported their introduction in the public sector. Knowledge was poor, inconsistent attitudes, and they felt their ability to meet the demand of potential users is limited.
(Thapa 2013)	Nepal: Kathmandu and Lalitpur district	Descriptive quantitative survey	60 Nurses: ANM 29,	To examine knowledge, attitude and practices of EC among nurses	96.33% of nursing personnel had knowledge on general information of Emergency Contraception (EC), 88.78% had knowledge about intrauterine contraceptive device as EC, 66.1% had knowledge on general information of emergency contraceptive pills, 65.5% had knowledge on its dosage and administration and only 59.05% had knowledge on its side effects and their management. On an average, 72.83% of them had knowledge on EC as a whole. More than three- fourth (78.18%) of them had positive attitude towards EC. When comparing nurses’ knowledge between educational qualifications, training on EC, duration of experience and between in-service training on family planning counselling, there was statistically no significant difference on knowledge between these variables. When studying the correlation between nurses’ knowledge and attitude regarding EC, it was found to be moderately correlated (r = 0.537)
			Staff Nurse 17, Senior Staff Nurse 11, charge nurse 3		
(Tripathi, Rathore & Sachdeva 2003)	India:	Descriptive quantitative survey	Two groups of clients (abortion seekers at Family Welfare centre, and non-medical college students (prospective clients)); and 4 groups of health care providers (gynaecologists, general practitioners, paramedical workers, and medical students)	To describe knowledge, attitude, and practices among health care providers	Few of the clients were familiar with the concept of emergency contraception and so the rest of the information could not be obtained from them and hence this was excluded from further analysis. Many providers (84.8% gynaecologists, 41.0% general practitioners, 2.7% paramedical workers, and 64.4% medical students) were vaguely familiar with the concept of emergency contraception, very few knew accurately about timing and doses. The majority of these thought it to be an essential component of contraceptive services but preferred distribution through health care providers only. The practice of emergency contraception as reported in the present survey was inconsistent. Yuzpe regimen was the most commonly used method and nausea/vomiting were the commonest side-effects. The question of efficacy of emergency contraception was not answered reliably by the health care providers.
(Worku & Teklu 2011)	Ethiopia: Addis Ababa	Descriptive quantitative survey	Drug dispensers ***(n = 303)*** working in pharmacies and drug stores of Addis Ababa	To describe EC Knowledge, attitudes and practices	84% of respondents (n = 256) were aware of EC, but the remainder had never heard of it. Although nearly 80% of aware respondents had a knowledge and attitude score of more than 50%, only 25% had knowledge and 35% had an attitude score of more than 75%. Amongst aware respondents, 32% had prescribed ECP.

### Provider knowledge

Many studies examined the knowledge of providers including: timing and administration of ECP; its mechanism of action; indications; eligibility; side effects; safety; effectiveness; sites at which it is available; cost; sources of information and factors associated with knowledge. In many of the papers, providers correctly identified at least one emergency contraception product available in their country [25-35]. However, in some countries, some respondents incorrectly identified other products as ECP. Medicine indicated for menstrual irregularities, and the malaria therapy (quinine) were erroneously noted as ECP by medical doctors [33] and drug vendors [36] in Nigeria.

Data concerning provider knowledge was available in some studies [25,28,31,34,37-41]. While most providers surveyed felt ECP was safe [28,35,42] more than half of participants in some studies held reservations [30,34,37,41,43,44].

### Provider attitudes and beliefs

A lack of understanding of the biological mechanism of action underpinning ECP was noted among many study respondents; some were not aware of the disruption of ovulation or said that ECP blocks the implantation of the fertilized ovum, or were not sure of the mechanism [30,42-44]. Some providers described ECP as an abortifacient [29,37,39,40,42,43,45-47]. In some studies in the review providers incorrectly believed that ECP use could result in congenital abnormalities if a woman was pregnant [29,35,37,39,40,43]. Infertility was a concern of some Nicaraguan study participants [37], while some Ethiopian pharmacists and drug vendors said that ECP could cause uterine and breast cancer, skin pigmentation, abdominal pain, and high blood pressure [35,39].

In some studies, providers expressed concern that ECP use might displace regular contraception use [25,30,35,37,39,47]. Others said they were confident that by counselling their clients they could prevent them from using ECP as a regular method. Health providers in some studies also expressed concern that increasing the availability of ECP would promote earlier sexual debut [35] and lead to unsafe sex [32,48], an increase in STIs and HIV [37,49].

Barriers to prescription and/or dispensing were noted in a few studies, including low consumer demand [26,36], and ethical, legal and religious concerns of the providers [34,36,43]. ECP were regarded by some as delivering a cost saving to the health system through reducing the abortion rate [41] and unwanted child bearing [32], as well as an opportunity to promote regular contraception [47].

Providers in one study were found to be more judgemental of clients who did not use family planning methods other than ECP, nevertheless, most providers said they still served such clients [47]. Some study participants felt that ECP should not be available to unmarried women and adolescents because access would encourage sexual activity [32,37].

### Provider practice

Data from the findings of 25 of the papers made reference to the practice of various providers in relation to their prescription or dispensing practices, or interaction with consumers. Four sub-themes emerged describing factors related to provider practice. These sub-themes are: volume of ECP sales, provider characteristics, client characteristics, information and advice. Only two of these studies provided observational data of provider practice from the perspective of “mystery clients” [50,51] while the reminder of the documents relied on provider recall and self-report.

In terms of volume of ECP sales, about a quarter of respondents in a Ghanaian study said they provided ECP to clients every day and another quarter provided it several times through the month [47]. In a Nigerian study when providers were asked the number of clients they supplied ECP with each month, the answer ranged from 0–45 women, more than 50% of the respondents served fewer than 10 clients per month [52]. The average estimate per pharmacy staff member in a study in Addis Ababa was 22.5 ECP clients per month [39]. This average was similar to drug vendors surveyed in Nigeria where about 43.2 per cent reported a clientele of fewer than 20 people monthly however, one quarter (25.3 per cent) had 20–100 clients, and six per cent had a 100 or more clients monthly [36]. One study noted that the number of packs sold in a month per pharmacy varied from two to 500 packs/month, with a mean of 62 packs every month [53].

ECP is offered over the counter in many countries and women are able to decide for themselves whether they need ECP. The safety profile of ECP, as well as the simplicity of the regimen means that many or most women do not require information when they purchase ECP, other than that provided with the package. However, some women may need or wish more information from providers. The likelihood of providing information and advice to clients purchasing ECP was reported in some studies. In one Nigerian study fewer than one in 10 providers said they always provided information to clients, while one-fourth never provided information to clients obtaining ECP [46]. Nine out of 10 Indian pharmacists surveyed reported simply providing ECP to customers [42]. Two studies involving the use of mystery clients who presented at various pharmacies found that those whose role involved playing an inexperienced client were significantly more likely to be offered additional information on ECP rather than when they presented as experienced users of ECP [50,51], suggesting that providers have the capacity to correctly judge when it is appropriate to offer women purchasing ECP the opportunity to receive more information.

Providers in a number of studies noted a range of ECP consumer characteristics [36,41,42]. The providers interviewed in Khan et al. believed that unmarried younger women or students are more likely to go to pharmacies, whereas married older women are more likely to go to other providers; younger, unmarried women are least likely to go to qualified doctors [42].

Various provider characteristics were examined in some research to identify the factors associated with the provision of ECP including provider knowledge, gender, and place of work, religion, experience and profession. Busy retail outlets were said to limit the provision of information and counselling [50] and stock outs affected provider’s ability to dispense [36,42,47,51,52].

Police procedure for processing women who have been sexually assaulted was described by Ethiopian police interviewees in the Kassa et al. study [54]. Women were said to present first at a police station after being assaulted because they require police certification as evidence of assault before being able to receive treatment. A female police officer responsible for dealing with such crimes would interview the victim, file a report and then refer the women to a health facility, usually one that would provide a medical report in a short time frame to aid the investigation [54].

### Provider education and training

Providers identified ECP training as a need in four studies [25,32,52,54]. Some surveyed Nigerian and Nepalese providers reporting having received ECP training [41,45] while one Senegalese study found that a considerable proportion of providers (38%) had not received any ECP training [49]. Four studies described training initiatives that had led to improvements in provider knowledge and dispensing practice and more positive attitudes towards ECP [28,47,55,56]. A study of ECP from Guatemala included training for police officers, social workers and health professionals. The findings indicated that these professionals not only shared their ECP knowledge and practice experiences with colleagues in their own organizations but across these sectors [55].

## Discussion

This narrative synthesis of 37 documents provided insight into the knowledge, attitudes, practices and training of those who provide ECP to women and men in LMIC. The analysis of the findings of studies retrieved in this review revealed a focus on formal health workers and less on drug vendors, pharmacy staff, lay health workers and providers in the education, justice and social service sectors. This reveals gaps in knowledge about the role of private sector providers, which is particularly acute given the prominent role that the private commercial sector plays in making ECP widely available in LMICs and elsewhere in the world. In general, the literature on the pharmacy sector is scanty, posing a significant gap given that ECP are mostly provided in this sector in the majority of LMIC [57]. None of the papers reviewed focused on providers in humanitarian contexts [58]. Gaps are also visible in the role of health staff in setting such as youth clinics or in schools where adolescents can be provided access to ECP. There is also a dearth of literature reflecting the perspectives of providers in LMIC in the Western Pacific, South America and Central Asia.

### Improving human resources for health performance to deliver ECP

Evidence of workforce interventions to improve practice is scarce in the documents reviewed, particularly in terms of the types of workforce support and performance management initiatives that may be required across both the public and private sectors to deliver ECP. Given the number of studies identifying provider knowledge gaps, less than favourable attitudes and issues with practice, investigations into approaches to optimize provider performance to deliver ECP may be useful. This could involve the development of workforce initiatives for the provision of ECP that could be assessed against outcome measures in key human resources for health (HRH) areas such as: policy, regulation and legislation; management systems; education, training and competencies and; consumer engagement. These areas have been described as useful fields for guiding the improvement of workforce performance in reproductive health [59] and are discussed below in relation to indictors for increasing access to ECP. The development and implementation of performance indicators alongside training and structured supervision may not be feasible in context of small, informal or sole operator businesses selling ECP. Other mechanisms for supporting providers in the retail setting, such as pharmaceutical “detailing” or short-course format training, may be more feasible and could possibly play an important role in strengthening access to ECP. However, these alternative strategies, while already in use by social marketing organizations and commercial pharmaceutical distributors, have not been evaluated and do not appear in the published literature reviewed here. More formal performance management interventions may still play a role as they could potentially be usefully applied to pre-service training, and to larger chain retail pharmacies and franchised outlets [60] that have experienced rapid growth in LMIC such as India [61].

#### Workforce and ECP Policy, regulation and legislation

We were not able to identify research concerned with national and district HRH policies that address health workers in the public, private and non-state sectors, job classification systems, registration, certification, or licensing requirements or service functions [62]. While the movement of ECP from a prescription to an over-the-counter (OTC) drug has been a crucial step in expanding access, it appears from our review that nothing exists in the formal literature examining or documenting the impact of this policy change. Future research may be useful to investigate how workforce policy is, or can be best aligned with national reproductive health policies, plans and laws to facilitate access to ECP and how the changing patterns of provision of ECP, from a prescription product in clinic settings to a OTC drug in commercial settings has affected training and information needs for these new cadres of providers. The development of national policy targets in monitoring and evaluation of HRH plans for example may be helpful for measuring progress including in terms of ECP provision. Indicators in this area could include the proportion of workers trained in ECP and if appropriate, the numbers of providers licensed to provide ECP.

#### Management systems

HRH management refers to staff supply (staff numbers, skills mix and workforce planning), performance management (supervision and motivation), and personnel administration and employee relations (pay and incentives) [63]. There appears to be a lack of research examining the distribution of personnel who dispense ECP in both the public and private health sectors and beyond, and a major gap in the literature regarding the transition of ECP provision from public to private/commercial sector and the impact . This data would provide valuable information for future planning workforce and co-ordination to ensure efficient use of current staff.

Although nurses and midwives were included in the generic studies of health providers in the review only one study focused on nurses. The role of nurses and midwives in reproductive health provision is central and as they comprise the majority of health workers in most countries and it has been argued that the role of mid-level cadres deserves particular emphasis in LMIC where there are chronic shortages of staff [7,64]. Task shifting and task sharing practices involving the delegation of reproductive tasks from doctors to nurses and midwives has been found elsewhere to show promise [60,65,66]. In addition shifting tasks relating to the distribution of ECP to paramedical and community health workers may be a useful strategy as has been found in other research [67]. However these cadres will require training and supportive supervision to carry out these new duties. The current literature does not explore how midwives and nurses can support ECP knowledge (for instance, during contraceptive counselling, post-partum education, etc.) even if they are not typically dispensers of ECPs after the transition to a commercial sector, pharmacy environment.

Research concerning the actual performance of ECP providers other than self-report was lacking in the review, as were standard measures to assess outputs. Calls have been made for measurements of quality in the sexual and reproductive health workforce, in order to provide incentives to deliver quality care and pay for performance [68]. Performance indicators could be developed to include ECP and applied at both health service and provider levels and in the commercial sector. The assessment of provider performance indicators would need to involve quality audit and supervisory processes both of which have been shown to contribute to improve health worker performance and health outcomes [69]. Supervision is also an important ancillary aspect of paying health workers financial incentives for performance that is in line with public health goals [70]. However lacking in the review is research concerning the best practices in providing financial and non-financial incentives to workers to ensure the availability of ECP in the public and private sectors. Vouchers for vulnerable groups may improve access to contraception including ECP [71] but the evidence for pay for performance incentives in LMICs is not strong [72]. It may be that in the commercial sector, sales of ECP at a profitable price can generate adequate motivation for providers to offer ECPs free of barriers however, we did not discover any literature that explores the issue of profit in motivating workforce.

Quality staff performance requires bundles of intervention [73]. This includes audit and supportive supervision in combination with a range of other initiatives such as incentives, job aides and training [69]. Research in high income countries may be transferable to LMIC contexts to improve provider performance to dispense ECP. In Australia, a job aide in the form of a written checklist was found to improve the quantity and consistency of patient assessment but not the quality of advice [74]. Some countries have developed practice guidelines [75] that may provide a direction for others wishing to better support clinicians.

#### Education and training

In this review no studies were found that examined the pre and in-service curriculum of health providers as it pertained to ECP. This concurs with other studies that have found few evaluations of training initiatives for health and human services providers [76]. Knowledge of useful education and training interventions would help to identify gaps and inconstancies, align curricula with national policy and integrate interprofessional learning opportunities where appropriate. The need for professional education and training has been noted particularly in the commercial sector where turnover of retail staff is high [11]. This need is being partially addressed by the WHO, the United Nations Population Fund (UNFPA) and the United States Agency for International Aid (USAID) Training Resource Package for Family Planning. This package offers essential resources including separate modules for ECP for family planning and reproductive health trainers, supervisors, and program managers (http://www.fptraining.org./). Insight into education efforts to address judgemental attitudes towards particular consumers and ECP related stigma could be gained from mental health and pharmacy education where contact-based education was found to reduce mental illness-related stigma [77].

Few studies captured data on the information and advice given to consumers. While in some contexts, information provision may not be necessary as women may have prior knowledge or prefer to read the packet, asking consumers if they wish to have further information would provide an opportunity to offer this if required. While encouraging results were found in two studies [50,51] suggesting that providers could ascertain when women required information, training to hone these skills and best communicate the information women want may be useful.

Four studies in the review included exploration of the knowledge, attitudes and practices of non-health personnel and as previously noted there is little emphasis on private sector providers. Lessons from interventions focused at retailers in other areas such as malaria may be useful. A review of medicine retailers selling malaria medications in sub-Saharan Africa found that on-going supervision and on-the-job-training can contribute to improving the appropriateness of drugs and information provided by these providers [78]. One study in the current review found that training providers in the health, community and justice sectors has positive outcomes in knowledge gains, attitudes and self-reported practice [55]. More knowledge is needed to better understand best practices in improving and training the ECP-capacity of different cadres across sectors. Professional collaboration between health workers and the police has been found to improve access to ECP, however this was the result of a number of initiatives beyond training, including changing policies to enable police officers themselves to provide ECP and introducing reliable supplies, supportive supervision, and revised protocols [13]. Collaboration across the education and community sectors may also be possible through such supportive workplace environments. There is evidence in primary health care contexts that collaboration across sectors can contribute to positive intermediate changes in health knowledge, attitudes, behaviour, and in the environment through new policies, practices and services [79].

#### Community and consumer engagement

The primary health care approach [80] acknowledges the resourcefulness of communities and the importance of community participation in their own health care. Provider engagement with the community to build relationships, understand and best respond to needs is therefore necessary to increase access to ECP. Provider performance in this area could be gauged through measures of client satisfaction, consumer/patient contacts, and the presence of a formal relationship with community organisations and leaders.

Collaborative community based social marketing initiatives [81] and education could engage community members, consumers and providers in positive ways to promote ECP. Participatory women's groups might improve prove effective in disseminating information about ECP and preventing unwanted pregnancy as they have in the improvement of maternal and neonatal survival [82]. Partnerships with community members to distribute ECP may be beneficial alongside strengthened health system logistics to ensure equitable access. Lessons from other drug distribution efforts may prove useful such as the effective delivery of Intermittent Preventive Treatment of malaria during pregnancy (IPTp) through traditional birth attendants, drug shop vendors, community reproductive health workers and adolescent peer mobilisers in Uganda [83]. However community members who distribute health commodities are also driven by their own personal agendas and interests that can be at odds with public health goals. For example community distributors and drug vendors may have preconceived views about those that seek ECP and how often they wish to procure it, or the stigmatization of certain groups such as unmarried women and adolescents that may affect women’s access to ECP. Knowledge of distributors’ motivations can help to provide more realistic expectations from such programmes and improve relationships between community distributors, consumers and formal providers [84].

### A model for monitoring performance

If provider performance is to be improved in order to increase access to ECP then decisions may need to be made concerning the indicators that should be used to assess such performance and how they need to be tailored to the diverse provider contexts. Workforce performance may need to be considered in relation to other aspects of health systems strengthening included in the WHO health systems framework for action (WHO 2007) and alongside health targets such as Millennium Development Goal (MDG) 5b concerning universal access to reproductive health. The perspective therefore needs to be multidimensional and involve the development of input, process and output level indicators [85] to link planning, implementation and evaluation aspects of ECP provision. This will ideally include the collection of quantitative data concerning the number of ECP actually sold or dispensed, as well as contextual information concerning stocks and supply. However, sales data from the commercial sector are generally not available and therefore it is difficult to ascertain how much ECP is being distributed in a certain country. Some market research data is available for a fee for countries including Cameroon and Kenya [86]. The best proxy for the distribution of ECP may be reports of ECP knowledge and use in population-based surveys such as the Demographic and Health Survey [87] or the Performance Monitoring and Accountability 2020 [88] surveys. Table 3 provides some examples of indicators at input, process and output levels across the six building blocks of the WHO health systems framework. Plotting indicators in a matrix format can provide an opportunity to see how indicators in each of the areas might relate so that appropriate measures can be selected for assessing provider performance.

**Table 3:** **Examples of performance indicators alongside others in the health system related to reproductive health**

Workforce	Service delivery	Finance	Leadership and governance	Information systems	Supply of medical products, vaccines and technologies
Input level					
Percentage of trainees provided with knowledge and skills on ECP in a given year	Number of service delivery points offering ECP.	Percentage of public and private sector expenditures on ECP.	Existence of national population and reproductive health policy and plans.	Percentage of women 15–49 years currently using modern methods of contraception (and ECP) included in routine data collection	Percentage of service delivery points stocked with ECP according to needs
Process level					
Proportion of providers trained in ECP	Percentage of clients given counselling on ECP during a year	Proportion of government budget spent on ECP	Use of national policy targets in monitoring and evaluation of plans at all levels.	Collection of data according to national protocols at all level.	Percentage of ECP e supplies that are wasted
Output level					
Percentage of providers trained in relation to the number of clients who received ECP counselling	Percentage of clients expressing satisfaction with the counselling services received	Cost of ECP in relation to average family income	Number of targets met/protocols followed as per policy	Number of routine data collections as per protocol in household surveys	Available number of ECP in relation to need

Selected Indicators adapted from [89].

#### Study limitations

This review may have been limited by an incomplete identification of research studies; however, efforts were made to search bibliographic databases, meta-indexes and the websites of international agencies working in the field. Additional documents were retrieved as a result however some may have been at a lower quality as not all studies were published in peer reviewed journals. The application of a narrative synthesis methodology to the results of the studies included in this review may have led to a reduction in detail particularly in terms of contextual factors that are relevant to the outcomes of interventions. However we made efforts to maintain detail in this review through rich textual descriptions of the study’s findings that provided a narrative across all studies. The graphical elements included in the analysis were useful for identifying patterns and the tabulation of findings enabled structured comparisons where pertinent.

## Conclusion

Adequate numbers of well-motivated, managed and competent providers are a critical part of delivering lifesaving commodities to reduce maternal death and provide access to reproductive health to achieve MDG 5 targets. As ECP provision increasingly shifts from the health care clinic sector to the pharmacy sector through changing policies regarding OTC status, there is an urgent need to understand more about who is now providing ECP in LMIC and what kind of training they have and what training would be required to improve performance. Ideally, it would be important to establish and apply evidence-based workforce strategies in an integrated manner to attain optimum performance to improve access to ECP, for a wide range of cadres of staff, including pharmacists and drug vendors. This review reveals considerable knowledge gaps concerning the performance of those who currently prescribe and/or dispense ECP. A focus on developing ways to best support this emerging workforce may improve practice and hence access to ECP for women.

## References

[1] Singh S, Sedgh G, Hussain R (2010). Unintended pregnancy: worldwide levels, trends, and outcomes. Stud Fam Plann.

[2] Sedgh G, Henshaw S, Singh S, Åhman E, Shah IH (2007). Induced abortion: estimated rates and trends worldwide. Lancet.

[3] Population Division U (2012). World Population Prospects: 2012 Revision.

[4] WHO (2004). Maternal Mortality in 2000: Estimates developed by WHO, UNICEF, and UNFPA.

[5] Mbizvo MT, Zaidi S (2010). Addressing critical gaps in achieving universal access to sexual and reproductive health (SRH): The case for improving adolescent SRH, preventing unsafe abortion, and enhancing linkages between SRH and HIV interventions. Int J Gynecol Obstet.

[6] PMNCH (2010). Support the Workforce. PMNCH Knowledge Summary 6.

[7] WHO (2012). From Evidence to Policy: Expanding Access to Family Planning Optimizing the Health Workforce for Effective Family Planning Services.

[8] UN (2012). UN Commission on Life Saving Commodities for Women and Children Commissioners’ Report September 2012.

[9] ICEC (2013). The Unfished Agenda: Next Steps to Increase Access to Emergency Contraception.

[10] Dawson A, Gray N, Howes T, Kennedy E, Ith P (2011). Human Resources for Health in Maternal, Neonatal and Reproductive Health at Community Level: A profile of Bangladesh. HRH Hub Working Papers.

[11] Westley E, Kapp N, Palermo T, Bleck J (2013). A review of global access to emergency contraception. Int J Gynecol Obstet.

[12] Brieger WR, Osamor PE, Salami KK, Oladepo O, Otusanya SA (2004). Interactions between patent medicine vendors and customers in urban and rural Nigeria. Health Policy Plan.

[13] Keesbury J, Zama M, Shreeniwas S (2009). The Copperbelt Model of Integrated Care for Survivors of Rape and Defilement: Testing the Feasibility of Police Provision of Emergency Contraceptive Pills. In.

[14] Pacqué-Margolis S, Cox C, Puckett A, Schaefer L (2013). Exploring Contraceptive Use Differentials in Sub-Saharan Africa through a Health Workforce Lens. Technical Brief 11.

[15] UN: Technical Reference Team Commodity: Emergency Contraception [http://www.everywomaneverychild.org/images/content/files/trt/Emergency_Contraception_Final.pdf]. New York: United Nations; 2013.

[16] Popay J, Roberts H, Sowden A, Petticrew M, Arai L, Rodgers M (2006). Guidance on the Conduct of Narrative Synthesis in Systematic Reviews. A product from the ESRC methods programme.

[17] CRD (2009). Systematic Reviews CRD’s Guidance for Undertaking Reviews in Health Care.

[18] Moher D, Liberati A, Tetzlaff J, Altman DG (2009). Preferred reporting items for systematic reviews and meta-analyses: the PRISMA statement. Annals of internal medicine.

[19] NHS: Critical Appraisal Skills Programme (CASP) making sense of evidence 10 questions to help you make sense of qualitative research [http://www.sph.nhs.uk/sph-files/casp-appraisal-tools/Qualitative%20Appraisal%20Tool.pdf]. London: Public Health Resource Unit, National Health Service; 2006.

[20] Pluye P, Gagnon M, Griffiths F, Johnson-Lafleur J (2009). A scoring system for appraising mixed methods research, and concomitantly appraising qualitative, quantitative and mixed methods primary studies in Mixed Studies Reviews. Int J Nurs Stud.

[21] AACODS checklist [http://dspace.flinders.edu.au/dspace/]

[22] Nalwadda G, Mirembe F, Tumwesigye NM, Byamugisha J, Faxelid E (2011). Constraints and prospects for contraceptive service provision to young people in Uganda: providers’ perspectives. BMC Health Serv Res.

[23] Balaiah D, Tapase P, Chauhan S, Puri C (2005). Awareness, knowledge and perceptions of emergency contraception among health care providers in and around Mumbai. 2nd Indian Association of Social Sciences in Health National Conference on "Globalization and Health Equity".

[24] Health systems [http://www.who.int/healthsystems/topics/en/]

[25] Byamugisha JK, Mirembe FM, Faxelid E, Gemsell DK (2007). Knowledge, attitudes and prescribing pattern of emergency contraceptives by health care workers in Kampala, Uganda. Acta Obstetricia et Gynecologica Scandinavica.

[26] Tripathi R, Rathore AM, Sachdeva J (2003). Emergency contraception: knowledge, attitude, and practices among health care providers in North India. J Obstet Gynaecol Res.

[27] Geidam A, Kullima A, Sadiq G (2010). Knowledge, attitude and provision of emergency contraception among health professionals in Borno state northern Nigeria. Int J Health Res.

[28] Kishore V, Misro MM, Nandan D (2010). Providers’ knowledge, attitude and dispensing practices of E-Pills in government dispensaries of south district in Delhi, India. Indian J Community Med.

[29] Ibrahim ZM, Ahmed MR, Shaaban MM (2013). Knowledge, attitude and practice of emergency contraception among health care providers in Ismailia, Egypt. Middle East Fertil Soc J.

[30] Chowdhury S (2013). Attitudes of obstetrics and gynaecology professionals towards provision of medical termination of pregnancy and Emergency contraception pill services in south Kerala, India.

[31] Onwuhafua PI, Kantiok C, Olafimihan O, Shittu OS (2005). Knowledge, attitude and practice of family planning amongst community health extension workers in Kaduna State. Nigeria J Obstet Gynaecol.

[32] Syahlul D, Amir L (2005). Do Indonesian medical practitioners approve the availability of emergency contraception over-the-counter? A survey of general practitioners and obstetricians in Jakarta. BMC Womens Health.

[33] Oriji VK, Omietimi JE (2011). Knowledge, attitude, and practice of emergency contraception among medical doctors in Port Harcourt. Niger J Clin Pract.

[34] Worku H, Teklu S (2011). Knowledge, attitudes and practices (KAP) regarding emergency contraception among drug dispensers working in retail outlets of Addis Ababa. Ethiop Med J.

[35] Dawit A, Van der Merwe M, Smith J (2010). Emergency contraception: practice of service providers in Addis Ababa, Ethiopia. Afr J Nurs Midwifery.

[36] Fayemi MM, Oduola OL, Ogbuji QC, Osinowo KA, Oyewo AE, Osiberu OM (2010). The knowledge of emergency contraception and dispensing practices of patent medicine vendors in South West Nigeria. J Public Health Policy.

[37] Ehrle N, Sarker M (2011). Emergency contraceptive pills: knowledge and attitudes of pharmacy personnel in Managua, Nicaragua. Int Perspect Sex Reprod Health.

[38] Judge S, Peterman A, Keesbury J (2011). Provider determinants of emergency contraceptive counseling and provision in Kenya and Ethiopia. Contraception.

[39] Lemma DA: Emergency contraception in Addis Ababa: practice of service providers. Master of Public Health Thesis Pretoria: University of South Africa; 2009. Available at http://uir.unisa.ac.za/bitstream/handle/10500/3215/dissertation_lemma_d.pdf?sequence=1

[40] Mir AS, Malik R (2010). Emergency contraceptive pills: exploring the knowledge and attitudes of community health workers in a developing Muslim country. N Am J Med Sci.

[41] Thapa B (2013). Knowledge and attitude regarding emergency contraception among nursing personnel. J Chitwan Med Coll.

[42] Khan ME, Bhatnagar I, Varma DS, Dixit A (2012). Providers’ and Key Opinion Leaders’ Attitudes, Beliefs, and Practices Concerning Emergency Contraception in India: Final Report.

[43] Abdulghani HM, Karim SI, Irfan F (2009). Emergency contraception: knowledge and attitudes of family physicians of a teaching hospital, Karachi, Pakistan. J Health Popul Nutr.

[44] Creanga A, Schwandt H, Danso K, Tsui A (2011). Knowledge about emergency contraception among family-planning providers in urban Ghana. Int J Gynecol Obstet.

[45] Ahonsi B, Salisu I, Idowu A, Oginni A (2012). Providers’ and Key Opinion Leaders’ Attitudes, Beliefs, and Practices Concerning Emergency Contraception in Nigeria: Final Survey Report. Program Brief.

[46] Ebuehi OM, Ebuehi OAT, Inem V (2006). Health care providers’ knowledge of, attitudes toward and provision of emergency contraceptives in Lagos, Nigeria. Int Fam Plan Perspect.

[47] Mayhew S, Osei I, Bajos N (2013). Provider attitudes to emergency contraception in Ghana and Burkina Faso. Population (English Edition).

[48] Kassaye T, Dwizedi A (2009). Seeking ways in improving promotion and provision of emergency contraception in Addis Ababa hospitals. Ethiop J Reprod Health.

[49] Mané B, Brady M, RamaRao S, Bintou Mbow F (2012). Providers’ and Key Opinion Leaders’ Attitudes, Beliefs, and Practices Concerning Emergency Contraception in Senegal: Final program Report. Program Brief.

[50] Liambila W, Obare F, Keesbury J (2010). Can private pharmacy providers offer comprehensive reproductive health services to users of emergency contraceptives? Evidence from Nairobi, Kenya. Patient Educ Couns.

[51] Obare F, Keesbury J, Liambila W (2009). The provision of emergency contraceptives in private sector pharmacies in urban Kenya: experiences of mystery clients. Afr Popul Stud.

[52] Okonofua FE, Omo-Aghoja LO, Hammed AA, Osazee K (2009). A survey of the knowledge and practices of emergency contraception by private medical practitioners in Nigeria. J Chin Clin Med.

[53] Mishra A, Saxena P (2013). Over-the-counter sale of emergency contraception: a survey of pharmacists in Delhi. Sex Med.

[54] Kassa M, Hiwot YG, Abdella A (2009). Barriers to accessing emergency contraception by victims of sexual assault in Addis Ababa Ethiopia. Ethiop J Reprod Health.

[55] Kestler E, Ramirez L (2004). Informing the Medical Community in Guatemala about Emergency Contraception.

[56] Gawade P, Salvi V, Mathur K, Mutalik N, Shinde A (2009). Training auxiliary nurse midwives and other paramedical staff in dispensing emergency contracptive pills. Nurs J India.

[57] Peters DH, Mirchandani GG, Hansen PM (2004). Strategies for engaging the private sector in sexual and reproductive health: how effective are they?. Health Policy Plan.

[58] Onyango MA, Hixson BL, McNally S (2013). Minimum Initial Service Package (MISP) for reproductive health during emergencies: time for a new paradigm?. Glob Public Health.

[59] Dawson A (2010). Towards a Comprehensive Approach to Enhancing the Performance of Health Workers in Maternal, Neonatal and Reproductive Health at Community Level: Learning from Experiences in the Asia and Pacific regions. Discussion paper 2.

[60] Patel L, Bennett T, Halpern C, Johnston H, Suchindran C (2009). Support for provision of early medical abortion by mid-level providers in Bihar and Jharkhand. India Reprod Health Matters.

[61] Lowe RF, Montagu D (2009). Legislation, regulation, and consolidation in the retail pharmacy sector in low-income countries. S Med Rev.

[62] Islam M (ed.): Health Systems Assessment Approach: A How-To Manual. Arlington, VA: U.S. Agency for International Development in collaboration with Health Systems 20/20, Partners for Health Reformplus, Quality Assurance Project, and Rational Pharmaceutical Management Plus, Management Sciences for Health; 2007.

[63] Dieleman M, Harnmeijer JW (2006). Improving Health Worker Performance: In search of promising practices.

[64] GHWA (2013). Mid-Level Health Workers for Delivery of Essential Health Services A Global Systematic Review and Country Experiences.

[65] Ivers LC, Jerome JG, Cullen KA, Lambert W, Celletti F, Samb B (2011). Task-shifting in HIV care: a case study of nurse-centered community-based care in rural Haiti. PLoS One.

[66] Paul M, Gemzell-Danielsson K, Kiggundu C, Namugenyi R, Klingberg-Allvin M (2014). Barriers and facilitators in the provision of post-abortion care at district level in central Uganda-a qualitative study focusing on task sharing between physicians and midwives. BMC Health Serv Res.

[67] Malarcher S, Meirik O, Lebetkin E, Shah I, Spieler J, Stanback J (2011). Provision of DMPA by community health workers: what the evidence shows. Contraception.

[68] Nothnagle M, Cappiello J, Taylor D (2013). Sexual and reproductive health workforce project: overview and recommendations from the SRH workforce summit, January 2013. Contraception.

[69] Rowe A, de Savigny D, Lanata CF, Victora CG (2005). How can we achieve and maintain high-quality performance of health workers in low-resource settings?. Lancet.

[70] Basinga P, Gertler P, Binagwaho A, Soucat A, Sturdy J, Vermeersch C (2010). Paying Primary Health Centres for Performance in Rwanda. Policy Research Working paper 5190.

[71] Meuwissen LE, Gorter AC, Kester ADM, Knottnerus JA (2006). Can a comprehensive voucher programme prompt changes in doctors’ knowledge, attitudes and practices related to sexual and reproductive health care for adolescents? A case study from Latin America. Trop Med Int Health.

[72] Witter S, Fretheim A, Kessy FL, Lindahl AK (2012). Paying for performance to improve the delivery of health interventions in low- and middle-income countries. Cochrane Database of Systematic Reviews.

[73] WHO (2006). Working Together for Health. The World Health Report 2006.

[74] Schneider CR, Gudka S, Fleischer L, Clifford RM (2013). The use of a written assessment checklist for the provision of emergency contraception via community pharmacies: a simulated patient study. Pharm pract.

[75] Emergency Contraception Guideline [http://www.esog.org.et/Emergency%20Contraception%20Guideline.htm]

[76] Colarossi L, Billowitz M, Breitbart V (2010). Emergency contraception education for health and human service professionals: an evaluation of knowledge and attitudes. Health Educ J.

[77] Patten S, Remillard A, Phillips L, Modgill G, Szeto A, Kassam A (2012). Effectiveness of contact-based education for reducing mental illness-related stigma in pharmacy students. BMC Med Educ.

[78] Goodman C, Brieger W, Unwin A, Mills A, Meek S, Greer G (2007). Medicine sellers and malaria treatment in sub-Saharan Africa: what do they do and how can their practice be improved?. Am J Trop Med Hyg.

[79] Adeleye OA, Ofili AN (2010). Strengthening Intersec-toral collaboration for primary health care in developing countries: can the health sector play broader roles?. J Environ Publ Health.

[80] WHO (1978). Declaration of Alma-Ata International Conference on Primary Health Care.

[81] McKenzie-Mohr D (2013). Fostering Sustainable Behavior: An Introduction to Community-Based Social Marketing, Third edn.

[82] Prost A, Colbourn T, Seward N, Azad K, Coomarasamy A, Copas A (2013). Women’s groups practising participatory learning and action to improve maternal and newborn health in low-resource settings: a systematic review and meta-analysis. Lancet.

[83] Mbonye AK, Bygbjerg IC, Magnussen P (2007). A community-based delivery system of intermittent preventive treatment of malaria in pregnancy and its effect on use of essential maternity care at health units in Uganda. Trans R Soc Trop Med Hyg.

[84] Kaler A, Watkins S (2001). Disobedient distributors: street-level bureaucrats and would-be patrons in community-based family planning programs in rural Kenya. Stud Fam Plann.

[85] Hornby P, Forte P (2002). Guidelines for Introducing Human Resource Indicators to Monitor Health Service Performance.

[86] Emergency contraception market research [http://www.euromonitor.com/emergency-contraception]

[87] The DHS Program Deomographic and Health Surveys [http://www.dhsprogram.com/]

[88] Performance Monitoring and Accountability 2020 [http://www.pma2020.org/about-pma2020]

[89] ESCAP (2003). Handbook on Reproductive Health Indicators.

